# Blockage of the adenosine A_2B_ receptor prevents cardiac fibroblasts overgrowth in rats with pulmonary arterial hypertension

**DOI:** 10.1007/s11302-023-09952-z

**Published:** 2023-07-05

**Authors:** Mafalda Bessa-Gonçalves, Bruno Bragança, Eduardo Martins-Dias, Adriana Vinhas, Mariana Certal, Tânia Rodrigues, Fátima Ferreirinha, Maria Adelina Costa, Paulo Correia-de-Sá, Ana Patrícia Fontes-Sousa

**Affiliations:** 1 Laboratório de Farmacologia e Neurobiologia, Departamento de Imuno-Fisiologia e Farmacologia/Centro de Investigação Farmacológica e Inovação Medicamentosa (MedInUP), Instituto de Ciências Biomédicas Abel Salazar, Universidade do Porto (ICBAS-UP), R. Jorge Viterbo Ferreira, 228, 4050-313 Porto, Portugal; 2 Departamento de Cardiologia, Centro Hospitalar Tâmega e Sousa, Penafiel, Portugal; 3 Departamento de Química, ICBAS-UP, Porto, Portugal

**Keywords:** Pulmonary arterial hypertension, Right ventricular remodelling, Monocrotaline rat model, Adenosine A_2B_ receptor, Cardiac fibroblasts, Viability/proliferation, Collagen production

## Abstract

Sustained pressure overload and fibrosis of the right ventricle (RV) are the leading causes of mortality in pulmonary arterial hypertension (PAH). Although the role of adenosine in PAH has been attributed to the control of pulmonary vascular tone, cardiac reserve, and inflammatory processes, the involvement of the nucleoside in RV remodelling remains poorly understood. Conflicting results exist on targeting the low-affinity adenosine A_2B_ receptor (A_2B_AR) for the treatment of PAH mostly because it displays dual roles in acute vs. chronic lung diseases. Herein, we investigated the role of the A_2B_AR in the viability/proliferation and collagen production by cardiac fibroblasts (CFs) isolated from RVs of rats with monocrotaline (MCT)-induced PAH. CFs from MCT-treated rats display higher cell viability/proliferation capacity and overexpress A_2B_AR compared to the cells from healthy littermates. The enzymatically stable adenosine analogue, 5′-*N*-ethylcarboxamidoadenosine (NECA, 1–30 μM), concentration-dependently increased growth, and type I collagen production by CFs originated from control and PAH rats, but its effects were more prominent in cells from rats with PAH. Blockage of the A_2B_AR with PSB603 (100 nM), but not of the A_2A_AR with SCH442416 (100 nM), attenuated the proliferative effect of NECA in CFs from PAH rats. The A_2A_AR agonist, CGS21680 (3 and 10 nM), was virtually devoid of effect. Overall, data suggest that adenosine signalling via A_2B_AR may contribute to RV overgrowth secondary to PAH. Therefore, blockage of the A_2A_AR may be a valuable therapeutic alternative to mitigate cardiac remodelling and prevent right heart failure in PAH patients.

## Introduction

Pulmonary arterial hypertension (PAH) is a severe cardiopulmonary disorder characterized by pulmonary vascular remodelling resulting in a progressive increase in pulmonary vascular resistance and, subsequent, increases in the right ventricle (RV) afterload [1]. If left untreated, PAH rapidly progresses, leading to RV failure and death. Although the initial impact of PAH primarily affects the pulmonary vasculature, the survival of patients with PAH is closely related to the maintenance of the RV function [1]. Despite current medical advancements, PAH still carries a poor prognosis, thus prompting the urgent need for novel molecular targets to prevent and/or control disease progression and, thereby, reduce mortality [2].

Currently approved medications for PAH primarily focus on the reduction of pulmonary vascular resistance [2, 3], but none of the available therapies directly targets RV remodelling underlying heart failure [3, 4]. Few successful attempts using miRNA-based therapies have been attempted, but these novel biotechnological approaches are far from being clinically approved for use in PAH patients [5, 6]. The RV pressure overload secondary to PAH causes adverse ventricular remodelling as a consequence of cardiac fibrosis associated with excessive deposition of extracellular matrix (ECM) proteins [7]. Albeit interstitial cardiac fibroblasts (CFs) are the main protagonists in cardiac fibrosis, their enrolment in PAH-induced RV dysfunction remains poorly investigated, thus opening new research opportunities to address this unmet medical condition.

The purine nucleoside adenosine is a ubiquitous regulatory molecule existing both inside and outside all living cells. Adenosine controls cell functions by acting via a family of four membrane-bound G protein-coupled receptors, A_1_AR, A_2A_AR, A_2B_AR, and A_3_AR [8]. Increasing evidence suggests that the low-affinity A_2B_AR-mediated cell signalling is deleterious for the progression of heart dysfunction secondary to lung diseases since this receptor blockade has protective effects (for a review, see, e.g., [9, 10]). In animal models of pulmonary hypertension, overexpression of the A_2B_AR has been associated with increased levels of remodelling agents (e.g., IL-6 and matrix metalloproteinases) and signalling molecules implicated in the pathogenesis of PAH, such as endothelin-1 [11].

Data in the literature suggests that adenosine regulates myocardial remodelling by interfering with CFs proliferation and differentiation [12–14]. Indeed, CFs are endowed with all adenosine receptor subtypes [15], with high amounts of A_2B_AR mRNA being produced by these cells compared to other adenosine receptor transcripts. This valuable information contrasts with the gap in our knowledge regarding the role of the adenosine A_2B_AR subtype on RV remodelling in PAH (for a review, see, e.g., [9, 16]). Herein, this study may contribute to elucidating this issue by evaluating the effect of A_2B_AR in cell viability/proliferation and type I collagen production by CFs isolated from the RV myocardium of monocrotaline (MCT)-induced animal model of PAH, which is the most widely used experimental model for studying PAH in rats.

## Materials and methods

### PAH rat model

Male Wistar rats (*Rattus norvegicus*; Charles River, RGD Cat. No. 13508588, RRID:RGD_13508588) weighting 180–200 g were housed in groups of three to four animals inside ventilated Double Decker (38 cm high) cages with enriched environment and access to food and water *ad libitum*. The room temperature (21 °C) was kept constant and a regular light (07.30–19.30 h)–dark (19.30–07.30 h) cycle was imposed. The animals were acclimatized to these conditions for at least 10 days before their assignment to the two experimental groups: control (CTRL) and MCT-treated animals. MCT-induced PAH was generated by a single subcutaneous injection of MCT (60 mg/kg; MCT group; Crotaline; Sigma-Aldrich, St. Louis, MO). Control rats were injected, at the same time, with an equal volume of sterile saline (CTRL group; NaCl 0.9%).

Twenty-one days after MCT or saline injection, the rats were euthanized by decapitation (Small Animal Decapitator, DCAP, World Precision Instruments) followed by exsanguination for heart tissues collection. Trained authorized personnel performed these procedures roughly between 9 and 10 a.m. in strict accordance with the recommendations of the European Convention for the Protection of Vertebrate Animals used for Experimental and Other Scientific Purposes (ETS 123), Directive 2010/63/EU, and Portuguese rules (DL 113/2013). The euthanasia method used in this study was privileged considering that (1) shared tissue usage is a common practice in our laboratory to reduce the number of experimental animals (“3R” principles), and that (2) functional neurochemistry assays are routinely performed in our laboratory using isolated nerve terminals from different parts of the rat brain, thus preventing usage of drugs (e.g., sedatives, general anesthetic drugs, and carbon dioxide), which might affect neuronal functions before sacrifice and compromise data interpretation.

### RV histopathology assessment

The right heart hypertrophy was determined by Fulton’s index as the RV to the left ventricle plus septum (RV/(LV+S)) mass ratio. RV samples collected for histological analysis were stored in buffered 10% formaldehyde. After the initial fixation step, samples underwent dehydration (using ethanol in increasing concentrations), diaphanization (using xylene), and impregnation in liquid paraffin (54 °C). Paraffin-embedded RV samples were properly oriented inside metal frames before cutting into sections of 4-μm thickness. Then, sections were stained with hematoxylin and eosin (H&E) or Picro-Sirius Red to evaluate cardiomyocyte cross-sectional area and interstitial fibrosis, respectively. For the cardiomyocyte cross-sectional area, transverse sections were digitally photographed (Olympus IX81, Tokyo, Japan) and blindly measured using the Cell F imaging software (Olympus, Tokyo, Japan). Fifty muscle fibers per animal (3–4 rats per group) were analyzed only considering nuclei-centered cells for the analysis of cardiomyocyte dimensions. Photomicrographs of longitudinal sections of the RV stained with Picro-Sirius Red were assessed using the ImageJ software® (version 1.8.0, U.S. National Institutes of Health, MD, USA) for automated analysis of collagen content, as previously described [17, 18]. Images were captured with a monochrome charge-coupled device camera using a 20× magnification objective. The extent of fibrosis was determined by calculating the percentage of the Picro-Sirius Red-stained area over the total microscopic field area using the same magnification for all samples. A minimum of four separate images obtained from different (non-overlapping) regions of the RV were captured per tissue section; four tissue sections were analyzed per animal. The results obtained per animal were averaged within the same group, CTRL and MCT, for subsequent statistical analysis.

### Immunofluorescence confocal microscopy of RV slices

After dissecting the RV-free wall, ventricular strips were accurately isolated, stretched, pinned flat onto cork slices, and embedded in Shandon cryo-matrix (Thermo Scientific) before being frozen in a liquid nitrogen-isopentane mixture. Cryosectioned 10-μm thickness slices of the RV were thawed and fixed in phosphate-buffered saline (PBS) containing 50% acetone and 2% paraformaldehyde (PFA). Following fixation, the slices were washed three times for 10 min each using PBS and incubated for 2 h with a blocking buffer containing 10% fetal bovine serum (FBS), 1% bovine serum albumin (BSA), and 0.3% Triton X-10 in PBS. After blocking and permeabilization, the sections were incubated overnight at 4 °C with anti-vimentin (1:150, mouse, DAKO, UK) and anti-A_2B_AR (1:50, rabbit, #AAR-003, Alomone Labs) antibodies made-up in a PBS-based incubation buffer containing 5% FBS, 1% BSA, and 0.3% Triton X-100. Following washout of the primary antibody with PBS (3 cycles of 10 min), tissue samples were incubated with species-specific secondary antibodies (Alexa Fluor 568-labelled anti-mouse and Alexa Fluor 488-labelled anti-rabbit; Molecular Probes, Invitrogen, USA) in the dark for 2 h, at room temperature. Finally, the glass slides containing the RV sections were mounted using VectaShield medium with 4′-6-diamidino-2-phenylindole (DAPI) to counterstain the nuclei (H-1200; Vector Labs). Observations were performed and analyzed using a laser-scanning confocal microscope (Olympus Fluo View, FV1000, Tokyo, Japan). The relative amount of vimentin-positive fibroblasts per RV tissue slice was assessed using the ImageJ software® (version 1.8.0, U.S. National Institutes of Health, MD, USA) with a similar strategy to that used for collagen quantification [17, 18]. A minimum of two separate images, captured from different (non-overlapping) regions of the RV, were obtained per tissue section using a 20× magnification objective. Four tissue sections were analyzed per animal. Statistical analysis was performed using four animals per treatment group, CTRL and MCT.

### Cardiac fibroblast cell cultures

#### Isolation of RV cardiac fibroblasts and cell culture conditions

Rat CFs were isolated from the RV of CTRL and MCT animal groups, 21 days after injecting MCT or saline (CTRL). The cells were obtained by the explant technique in which fibroblasts migrate from minced tissue and grow in a fibroblast growth medium [19]. The cells were cultured on 96-well plastic-bottom plates using DMEM medium supplemented with 15% FBS, 1% of amphotericin B, and 1% of penicillin/streptomycin, at 37 °C in a humidified atmosphere of 95% air and 5% CO_2_. The medium was replaced twice a week. Primary cultures were maintained until they reached near confluence (~3–4 weeks). Adherent cells were enzymatically detached using a 0.04% trypsin-EDTA solution containing 0.025% type I collagenase in PBS. The resulting cell suspensions were cultured and maintained under the same conditions as mentioned above. All the experiments were performed in the first subculture [13, 14, 20–22].

#### Cell viability/proliferation assay

Viability/proliferation studies were performed using the MTT assay as previously described [12–14]. Rat CFs were seeded in flat plastic-bottom 96-well plates at a density of 3 × 10^4^ cells/mL and cultured in supplemented DMEM as described before. Cell cultures were routinely monitored by phase contrast microscopy and characterized on days 1, 7, 14, 21, and 28. The MTT assay consists of the reduction of 3-[4,5-dimethylthiazol-2-yl]-2,5-diphenyltetrazolium bromide (MTT) to a purple formazan reaction product by metabolically viable cells. During the last 4 h of each test period, the cells were incubated with 0.5 mg/mL of MTT in the conditions referred to above. Then, the medium was carefully removed and decanted, and the stained product dissolved with DMSO before absorbance (A) determination at 600 nm using a microplate reader spectrometer (Synergy HT Multi-Mode Microplate Reader, BioTek Instruments). The results were expressed as A/well.

#### Type I collagen determination﻿

 Type I collagen determination was performed using the Sirius Red staining assay. Rat CFs were cultured following the same protocol as described for the viability/proliferation studies [12–14]. The staining protocol was adapted from a previous study [23]. The cell layers were washed twice in PBS before cells fixation with Bouin’s fluid for 1 h. The fixation fluid was removed by suction and the culture plates were washed by immersion in running tap water for 15 min. The culture dishes were then allowed to air-dry before adding the Picro-Sirius Red dye (Direct Red 80). The cells were stained with the dye for 1 h under mild shaking on a microplate shaker. To remove the non-bound dye, the stained cells were washed with 0.01 N HCl and then dissolved in 0.1 N NaOH for 30 min at room temperature using a microplate shaker. Optical density was measured at 550 nm using 0.1 N NaOH as blank [14, 23]. The results were expressed as A/well.

#### Immunofluorescence staining of cardiac fibroblasts

Rat CFs were seeded in chamber slides at a density of 2.5 × 10^4^ cells/mL and allowed to grow for 7 days (see, e.g., [12–14]). Cultured cells were fixed in 4% PFA in PBS for 10 min, washed 3 times in PBS (10 min each), and, subsequently, incubated with blocking buffer I (10% FBS, 1% BSA, 0.1% Triton X, 0.05% NaN_3_) for 1 h. Primary antibodies diluted in blocking buffer II (5% FBS, 1% BSA, 0.1% Triton X, 0.05% NaN_3_) were applied [mouse anti-porcine vimentin 1:250 (DAKO); goat anti-human DDR2 1:25 (Santa Cruz); mouse anti-human α-smooth muscle actin (SMA)-FITC 1:250 (Sigma); rabbit anti-human A_2B_AR (2nd extracellular loop, 36 kDa) 1:50 (#AAR-003, Alomone Labs)] and the slides incubated overnight at 4 °C. The cells were then washed 3 times in PBS 1× (10 min each). The donkey anti-mouse IgG Alexa Fluor 488 (1:1000), donkey anti-rabbit Alexa Fluor Donkey 488 (1:1000), and donkey anti-goat IgG Alexa Fluor 633 (1:1000) secondary antibodies (Invitrogen) were diluted in blocking buffer II and incubated in the dark for 2 h, at room temperature. A final wash step was performed with PBS and, then, the glass slides were mounted with VectaShield medium with DAPI (Vector Laboratories) and stored at 4 °C. Observations were performed and analyzed using a laser-scanning confocal microscope (FV1000, Olympus) [12, 14, 24, 25].

#### SDS–PAGE and Western blot analysis

CFs were harvested and homogenized in a lysis buffer composed of 50 mM Tris-HCl (pH 8.0), 150 mM NaCl, 0.5% sodium deoxycholate, 1% Triton-X-100, 0.1% SDS, and a protease inhibitor cocktail. Protein quantification was assessed using the BCA protein assay kit according to the manufacturer’s instructions (Pierce, Rockford, IL, USA). Equal amounts of protein per sample (150 μg/lane) were resolved by SDS-PAGE under reducing conditions (i.e., the samples were solubilized in SDS reducing buffer containing 0.125 mM Tris-HCl, 4% SDS, 0.004% bromophenol blue, 20% glycerol, and 10% 2-mercaptoethanol, pH 6.8 at 70 °C for 10 min) using 10% polyacrylamide gels, and electrotransferred onto PVDF membranes (Millipore, MA, USA). The membranes were blocked for 1 h in Tris-buffered saline (TBS: 10 mM Tris-HCl, pH 7.5, 150 mM NaCl) containing 0.05% Tween 20 + 5% BSA, and then probed overnight at 4 °C with rabbit anti-human A_2B_ (second extracellular loop, 37 kDa) 1:200 (#AAR-003, Alomone Labs, Jerusalem, Israel) in the above-mentioned blocking buffer. Then, the membranes were washed three times for 10 min in 0.1% Tween 20 in TBS before their incubation with donkey anti-rabbit IgG (HRP) 1:70,000 (Abcam Plc, Cambridge, UK) secondary antibody for 120 min at room temperature. For normalization purposes, the membranes were also incubated with a rabbit anti-human α-tubulin (50 kDa) 1:2500 (#ab6046, Abcam). For the antibody specificity test, the rabbit anti-A_2B_AR primary antibody was pre-adsorbed with a 10-fold molar excess of its blocking peptide (#BLP-AR003, Alomone Labs, Jerusalem, Israel) corresponding to amino acid residues 147–166 of the human A_2B_AR second extracellular loop. After washing the membranes 3 times for 10 min, the antigen-antibody complexes were visualized by chemiluminescence with the Clarity Western ECL Substrate Kit (Bio-Rad Laboratories, Hercules, CA, USA) using the ChemiDoc MP imaging system (Bio-Rad Laboratories, Hercules, CA, USA). Gel band image densities were quantified with the ImageJ software (National Institute of Health, Bethesda, MD, USA).

#### Reagents and materials

Amphotericin B solution, penicillin-streptomycin, Direct Red 80 (Picro-Sirius Red 80, C_45_H_26_N_10_O_21_S_6_Na_6_), trypsin-EDTA solution, FBS, PBS, 5′-*N*-ethylcarboxamidoadenosine (NECA), 2-(2-furanyl)-7-[3-(4-methoxyphenyl) propyl]-7H-pyrazolo[4,3-e][1,2,4]triazolo[1,5-*c*]pyrimidin-5-amine (SCH442416), and 4-[2-[[6-amino-9-(nethyl-β-D-ribofuranuronamidosyl)-9H-purin-2-yl]amino]ethyl] benzene propanoic acid hydrochloride (CGS21680) were from Sigma-Aldrich (St. Louis, MO, USA). 8-[4-[4-(4-Chlorophenzyl)piperazide-1-sulfonyl)phenyl]]-1-propyl xanthine (PSB603) was from Tocris Cookson Inc. (Bristol, UK). Cell culture plates: 96-well plastic-bottom plates were purchased from Corning (New York, USA); cell culture chamber slides for confocal microscopy: glass-bottom chamber slides were purchased from Nunc (New York, USA). CGS21680, NECA, PSB603, and SCH442416 were diluted in dimethyl sulfoxide (DMSO); all other drugs were prepared in distilled water. Regarding solutions storage (as frozen aliquots at −20 °C) and dilution, pH control, and DMSO (maximum 0.05% v/v) testing, we followed that described in previous publications from our group [21, 22].

NECA (1–30 μM) and CGS21680 (3 and 10 nM) were used as enzymatically stable adenosine analogues to activate A_2B_AR and A_2A_AR in CFs, respectively. PSB603 and SCH442416 were used as potent and selective A_2B_AR and A_2A_AR antagonists, respectively. PSB603 displays <17,000-fold selectivity for A_2B_AR over other adenosine receptors [26], and SCH442416 displays >23,000-fold selectivity for A_2A_AR with minimal affinity for other adenosine receptors up to >10 μM concentrations [27].

### Presentation of data and statistical analysis

Data graphs and statistical analysis were carried out using the Graph Pad Prism 9.1.0 software (La Jolla, CA, USA). Data are expressed as mean ± SEM, unless otherwise noted, from an *n* number of individuals. No predetermined sample size calculation was performed. The D’Agostino–Pearson (omnibus *K*^2^) test was used to check for normality of data distribution; *p* < 0.05 values indicate that data passed the normality test. Student’s unpaired *t*-test or one-way analysis of variance (ANOVA), either corrected or uncorrected for multiple comparisons using the Bonferroni’s or the Fisher’s LSD tests, respectively, was applied only if *F* was significant and there was no variance inhomogeneity; few outliers were identified using the ROUT method with a *Q* = 1%. *p* < 0.05 (two-tailed) values were considered statistically significant.

## Results

### MCT-induced PAH model

**Fig. 1:**
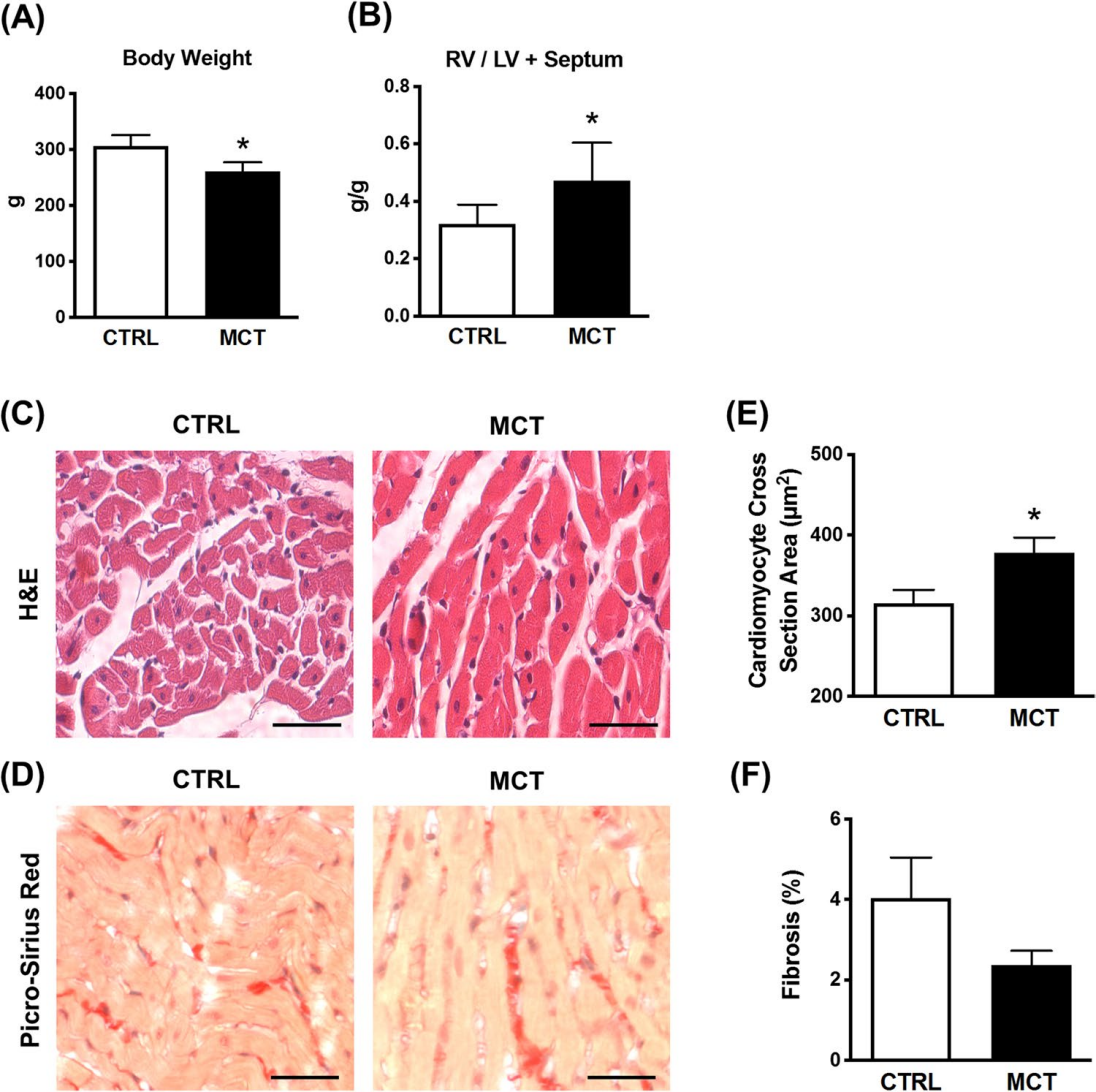
Histomorphometric analysis of the RV of rats with pulmonary arterial hypertension (PAH) induced by monocrotaline (MCT). Shown in **A** is the body weight and in **B** is Fulton’s index, which is the ratio between the mass of the RV over that of the LV plus the septum (RV/(LV+S)), of rats injected subcutaneously with saline (CTRL) or monocrotaline (MCT, 60 mg/kg) 21 days before the assessment. Tissue sections of the RV myocardium of both animal groups were stained with hematoxylin and eosin (H&E) (**C**) or Picro-Sirius Red (**D**). Scale bar: 50 μm. In **E**, shown is the average cross-section area (μm^2^) of at least 200 cardiomyocytes per animal group. In **F**, is represented the percentage of the Picro-Sirius Red positive staining (collagen deposition) to the total area of the micrograph, measured at least in 60 different tissue slices. Data are mean ± SEM, *n* = 5–9 animals/group. *p < 0.05 (Student’s unpaired *t*-test) represents significant differences compared to the CTRL group

As the disease progresses, the animals treated with MCT grew less (gained less body weight) than their control littermates; the body weight of MCT-treated animals was 15% less (*p* < 0.05) than control rats at sacrifice day, i.e., 21 days after MCT administration (Fig. 1A). MCT-treated animals exhibited marked RV hypertrophy indicated by the increase of Fulton’s index, i.e., the ratio between the mass of the RV over that of the LV plus the septum (RV/(LV+S)) (Fig. 1B). The histopathological analysis of tissue sections from MCT-treated rats revealed hypertrophy of RV cardiomyocytes (*p* < 0.05) compared to control littermates (Fig. 1C and 1E). These findings agree with data in the literature showing RV remodelling secondary to pulmonary pressure overload in animals with PAH [28]. However, at this disease stage, the RV of MCT-treated rats had no significant myocardial fibrosis as determined by the Picro-Sirius Red histochemical assay, which specifically identifies collagen deposition (Fig. 1D and 1F).

### Immunophenotypic characterization of RV cardiac fibroblasts

**Fig. 2:**
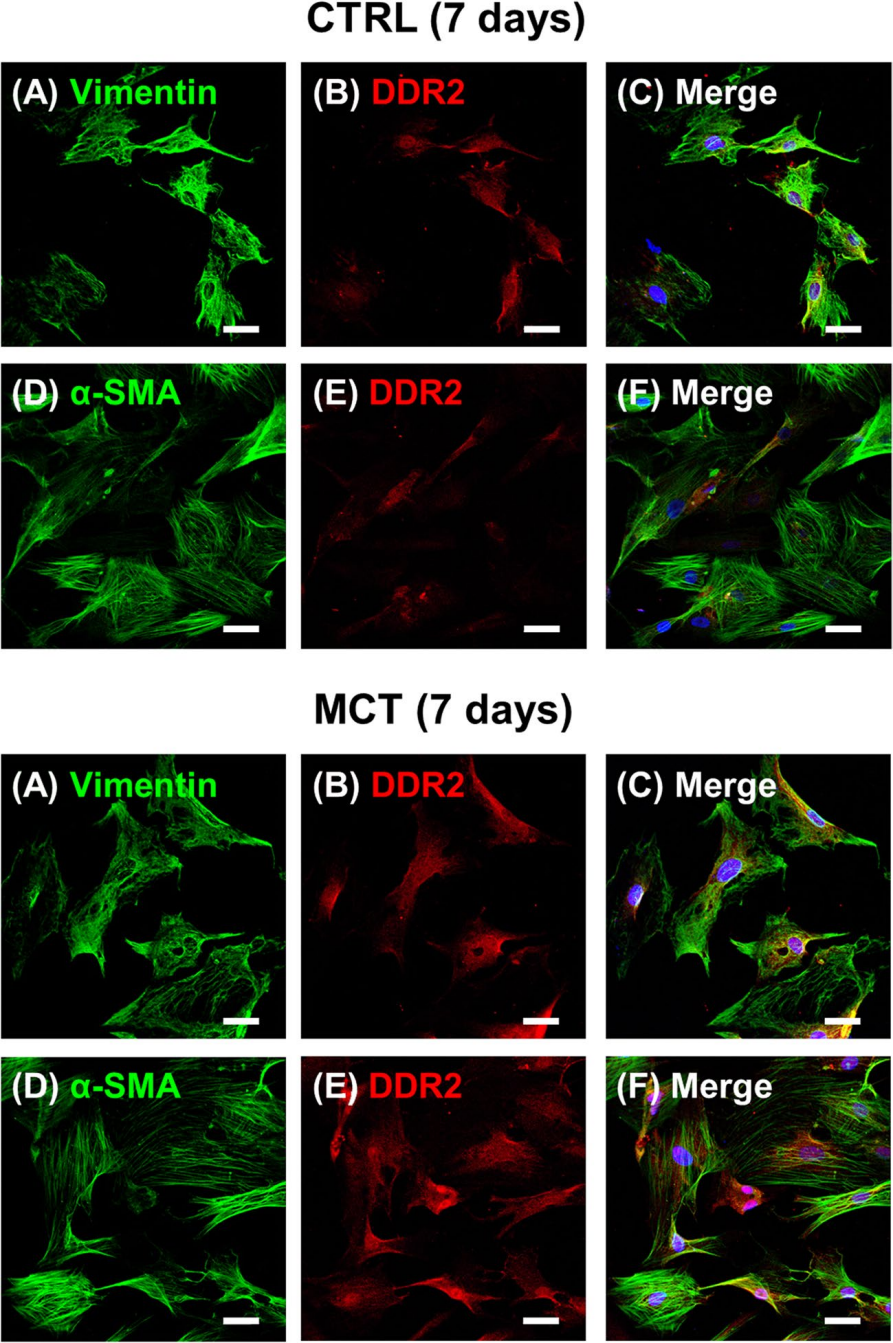
Immunophenotypic characterization of cardiac fibroblasts (CFs) isolated from the RV of CTRL and MCT-treated rats on culture day 7. Confocal microscopy images show that CFs exhibit positive immunoreactivity against vimentin (a reliable fibroblast marker; green, **A**), α-smooth muscle actin (α-SMA, a myofilament protein typically expressed by activated myofibroblasts; green, **D**), and discoidin domain receptor 2 (DDR2, a collagen-specific receptor tyrosine kinase specifically expressed in CFs and myofibroblasts; red, **B** and **E**). Panels **C** and **F** show a merge of vimentin and DDR2, and α-SMA and DDR2, respectively. Nuclei are stained in blue with DAPI; yellow denotes co-localization. Scale bar: 30 μm

CFs isolated from the RV of CTRL and MCT-treated adult rats displayed positive immunoreactivity against discoidin domain receptor 2 (DDR2) (Fig. 2B and 2E), a collagen-specific receptor tyrosine kinase, considered the most specific marker for CFs and myofibroblasts [29, 30]. The increase in DDR2 density suggests that CFs cultured from the RV of MCT-treated rats returned to an early activation status. Moreover, these cells also stain positively against vimentin (Fig. 2A), an intermediate protein filament considered a reliable fibroblast-cell marker [31], as well as against α-SMA (Fig. 2D), a myofilament protein that is typically expressed in activated fibroblasts (myofibroblasts) [32]. This staining pattern indicates that RV-originated CFs cultured under the present experimental conditions present an activated myofibroblast phenotype, which is the CFs lineage most commonly associated with myocardial remodelling [33].

### Cardiac fibroblasts from MCT-treated rats proliferative more than cells from healthy controls

**Fig. 3:**
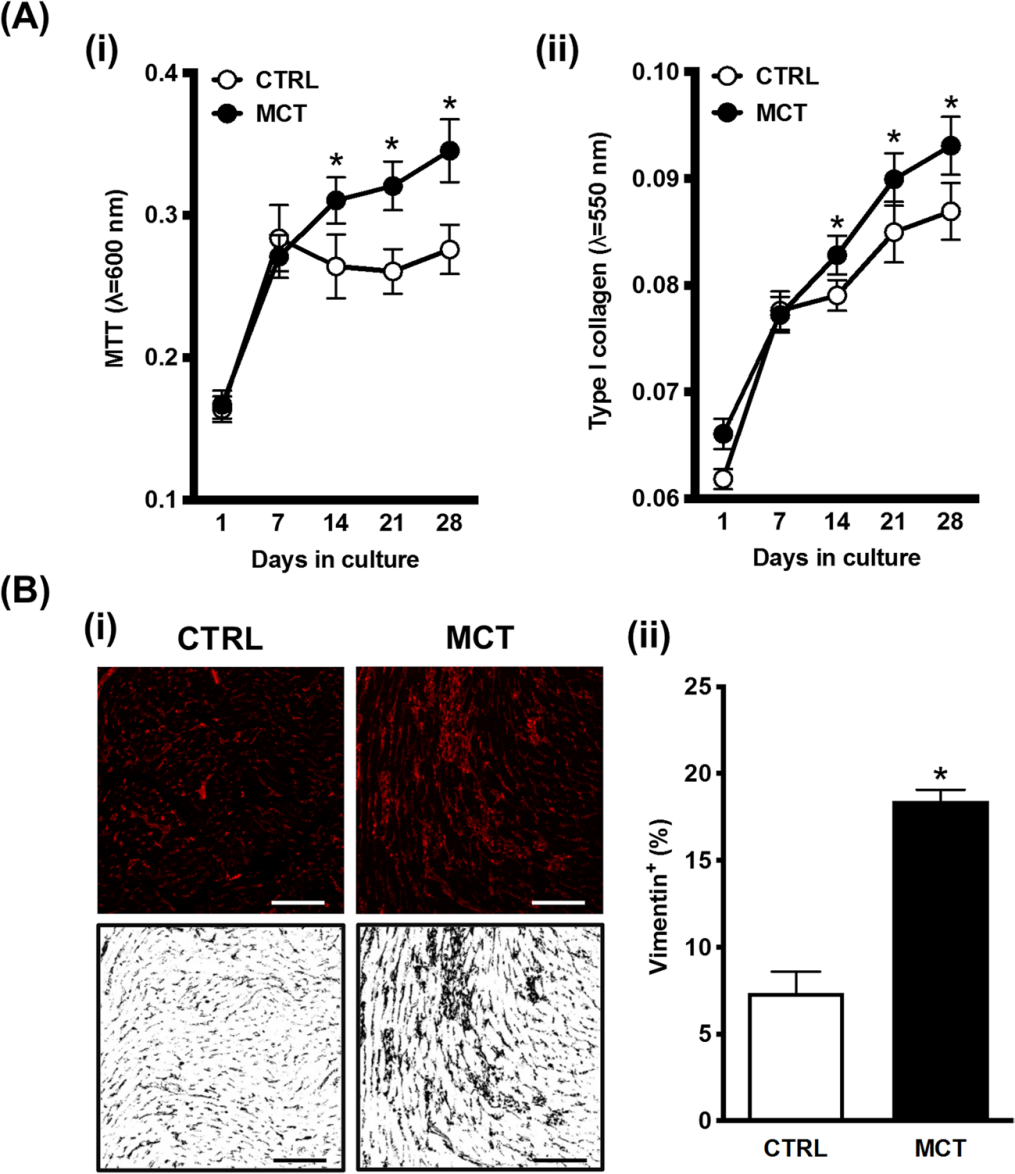
Cardiac fibroblasts (CFs) isolated from the RV of MCT-treated rats exhibit increased cell viability/proliferation (MTT assay) and produce higher amounts of type I collagen (Sirius Red assay) than cells from healthy controls. Panel **A** illustrates changes in viability/proliferation measured by the MTT assay (**i**) and type I collagen production assessed by the Sirius Red staining (**ii**) in CFs isolated from CTRL (*n* = 16) and MCT-treated (*n* = 18) rats. The ordinates represent absorbance determinations at 600 nm and 550 nm per well at certain time points (1, 7, 14, 21, and 28 days), for the MTT assay (**Ai**) and the Sirius Red staining (**Aii**), respectively. Data are mean ± SEM from an *n* number of individuals; the experiments were performed in triplicate. *p < 0.05 (ANOVA, one-way analysis of variance) compared to the CTRL group. In panel **B**, the red labelling in the upper panels represents vimentin-positive cells infiltrating RV interstitial spaces in CTRL and MCT-treated animals (**Bi**); scale bar: 100 μm. The binary (black and white) representation of the images (bottom panels) was used for automatic quantification by the ImageJ software® (version 1.8.0, U.S. National Institutes of Health, Bethesda, MD, USA) (**Bii**). Data are mean ± SEM; at least four individuals were analyzed per group. *p < 0.05 (Student’s unpaired *t*-test) represents significant differences compared to the CTRL group

Data in Fig. 3A show that CFs isolated from the RV of MCT-treated rats are metabolically more active and/or proliferate more (MTT assay) and produce higher amounts (*p* < 0.05) of type I collagen (Sirius Red assay) than the cells originated from their CTRL littermates. Interestingly, CFs from pressure-overloaded RV are more eager to reduce MTT (indicating increases in cell viability and/or proliferation; Fig. 3Ai) than to produce type I collagen (Fig. 3Aii) compared to the cells from healthy controls at this disease stage. In agreement with this theory, we observed a threefold (*p* < 0.05) increase in the density of vimentin-positive CFs in interstitial spaces of the RV obtained from MCT-treated rats compared to CTRL animals (Fig. 3B).

### Cardiac fibroblasts from MCT-treated rats overexpress the A_2B_AR

**Fig. 4:**
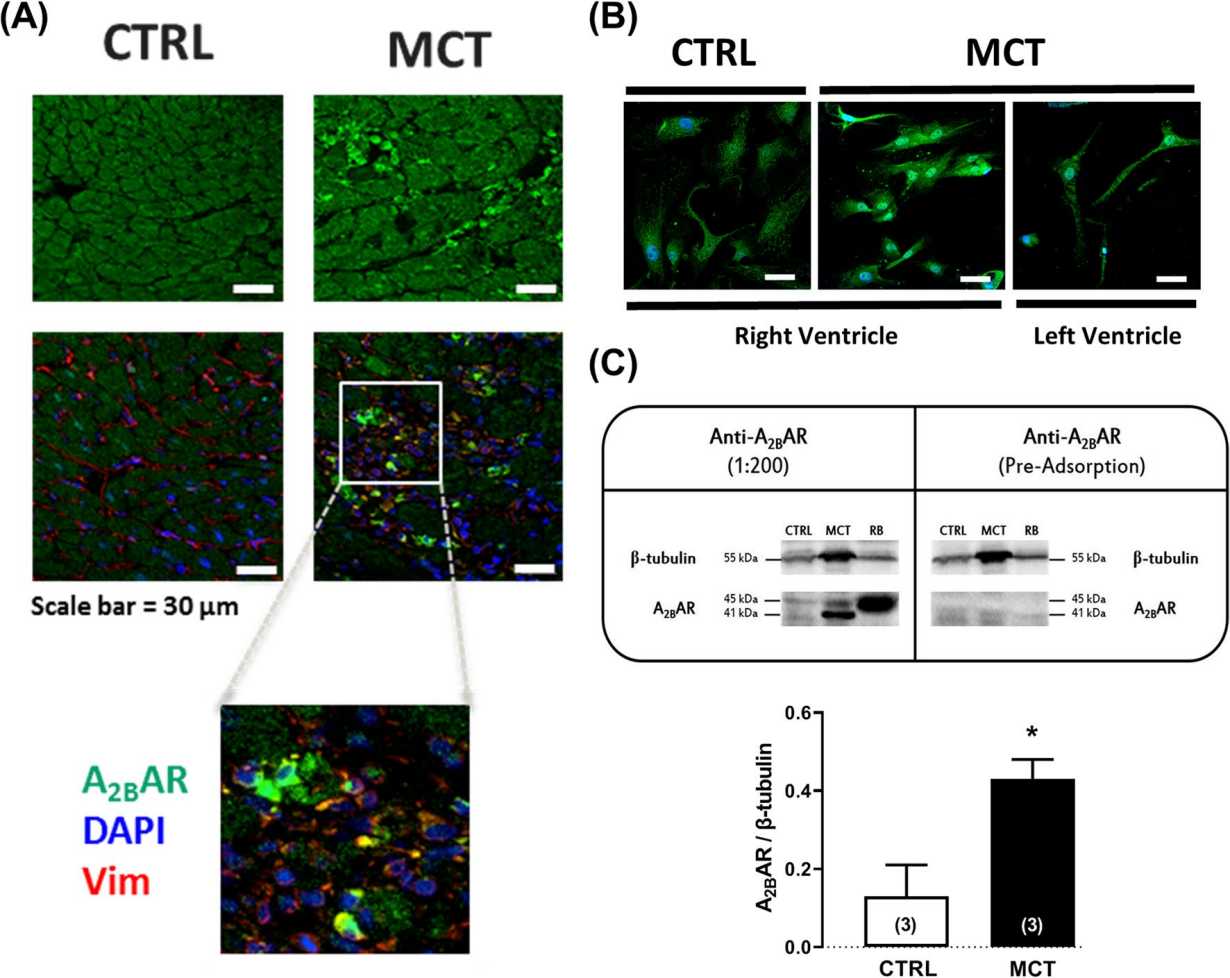
Cardiac fibroblasts (CFs) isolated from MCT-treated rats overexpress the adenosine A_2B_ receptor subtype. Shown are representative immunofluorescence confocal microscopy images of RV myocardium sections (**A**) and isolated CFs from the RV and LV myocardium (**B**) of CTRL and MCT-treated rats stained against A_2B_AR (green) and vimentin (red). Nuclei are stained in blue with DAPI. Micrographs are representative of at least five different individuals and were obtained with a laser-scanning confocal microscope using the same acquisition settings. Scale bar: 30 μm. In **C**, shown are representative immunoblots to document the relative amounts of the A_2B_AR in CFs isolated from the RV myocardium of CTRL and MCT-treated rats allowed to grow in culture for 28 days; gels were loaded with 150 μg protein amounts. Two protein species were recognized by the A_2B_AR antibody (#AAR-003, Alomone Inc., Jerusalem, Israel) corresponding to a peptide near the predicted molecular weight of the A_2B_AR (~37 kDa) and a higher molecular mass (~45 kDa) isotype. Please note that the naturally occurring A_2B_AR isotype is highly enriched in CFs isolated from the RV myocardium of MCT-treated rats compared to their CTRL littermates. Both bands fully disappeared after pre-adsorption of the A_2B_AR primary antibody with a 10-fold molar excess of the A_2B_AR blocking peptide (#BLP-AR003, Alomone Inc., Jerusalem, Israel) corresponding to amino acid residues 147–166 of the human A_2B_AR second extracellular loop (negative control). The rat urinary bladder (RB) was used as a positive control for the A_2B_AR. β-Tubulin (55 kDa) was used as a reference protein. Each bar represents pooled data from three different individuals; three replicas were performed in each experiment. The vertical bars represent SEM. *p < 0.05 (Student’s unpaired *t*-test) represents significant differences compared to the CTRL group

Using immunofluorescence confocal microscopy and Western blot analysis, we show here that CFs from MCT-treated rats overexpress the A_2B_AR compared to CTRL littermates (Fig. 4). In the RV myocardium of MCT-treated rats, the A_2B_AR immunoreactivity is more evident in interstitial spaces adjacent to hypertrophied cardiomyocytes, which display much weaker signalling against this receptor (Fig. 4A). The A_2B_AR-immunoreactive interstitial cell infiltrates comprise vimentin-positive CFs among other larger mononucleated cells (Fig. 4A), which are most probably mast and macrophage immunological cells similar to those overexpressing the A_2B_AR in the fibrotic human lung [34]. Notably, the A_2B_AR/vimentin double-immunolabelling significantly increases in the RV of PAH rats compared to their CTRL littermates (Fig. 4A). Likewise, we show here that CFs isolated from the RV of MCT-treated rats exhibit higher A_2B_AR-immunoreactivity levels than cells from their control littermates, but also when this comparison was made with CFs obtained from the left ventricle (LV) of PAH rats (Fig. 4B).

The confocal microscopy findings are strengthened by immunoblot analysis data showing that the A_2B_AR protein is upregulated in CFs isolated from the RV myocardium of MCT-treated rats compared to CTRL animals when these cells were allowed to grow for 28 days in culture (Fig. 4C). Interestingly, we detected two protein species: one close to the predicted molecular weight of the A_2B_AR (~37 kDa) and a higher molecular mass (~45 kDa) isotype. The naturally occurring A_2B_AR isotype is highly enriched in CFs isolated from the RV myocardium of MCT-treated rats compared to their CTRL littermates. Both bands completely disappear after pre-adsorption of the A_2B_AR primary antibody with a 10-fold molar excess of the A_2B_AR blocking peptide (#BLP-AR003, Alomone Inc., Jerusalem, Israel), which corresponds to amino acid residues 147–166 of the human A_2B_AR second extracellular loop (negative control).

### Activation of the A_2B_AR promotes the growth of cardiac fibroblasts from the RV of MCT-treated rats

**Fig. 5:**
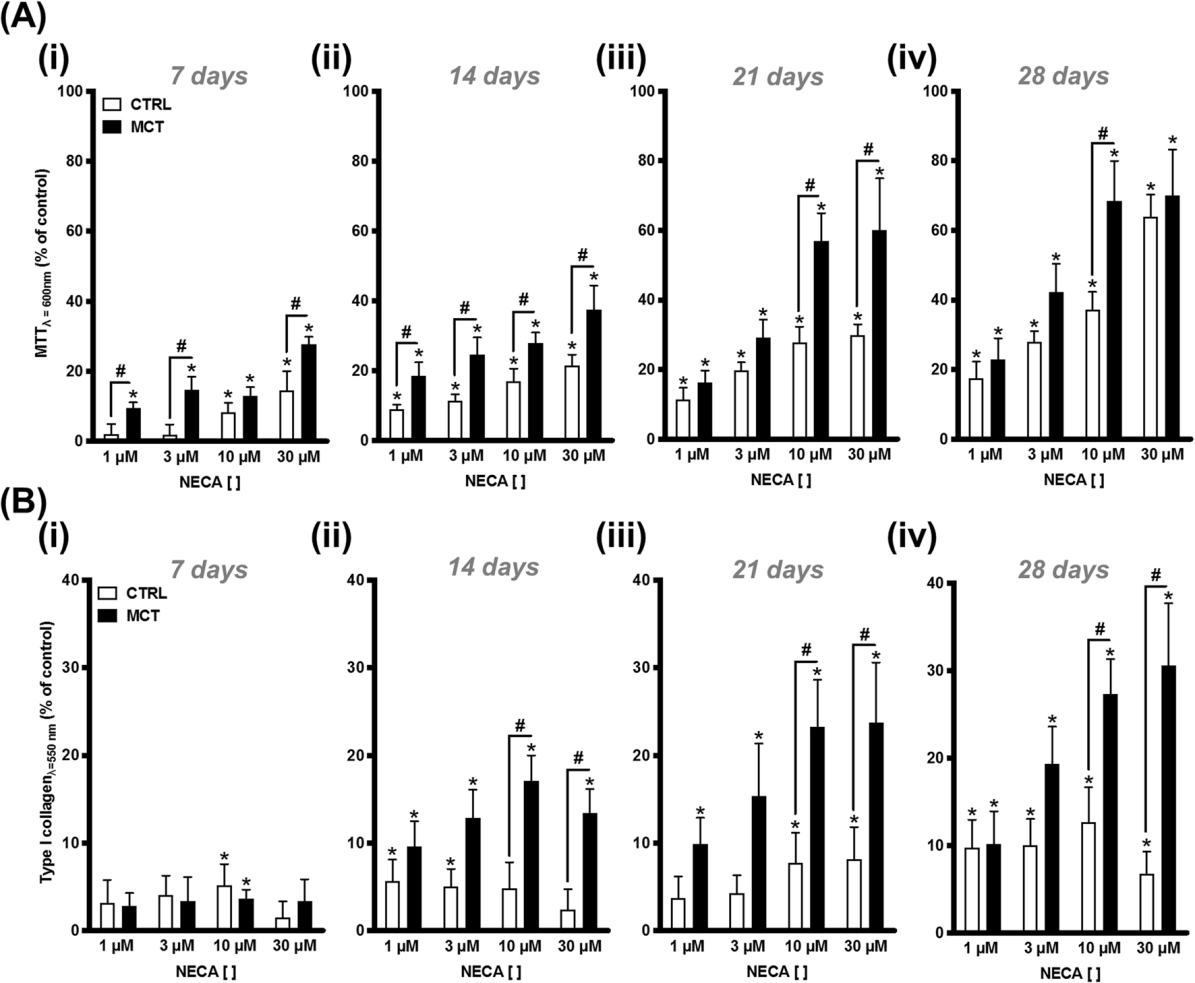
The enzymatically stable adenosine analogue, NECA (1–30 μM), concentration-dependently increases cell viability/growth and type I collagen production by cardiac fibroblasts (CFs) from the RV of CTRL and MCT-treated rats. The ordinates represent NECA (1–30 μM)-induced changes in cell growth (MTT assay, **A**) and type I collagen production (Sirius Red assay, **B**) compared to the control situation using the same cell batch in the absence of the adenosine analogue at culture days 7 (**i**), 14 (**ii**), 21 (**iii**), and 28 (**iv**). Zero represents the similarity between the two values (NECA vs. control); positive and negative values represent facilitation or inhibition of either cell growth or type I collagen production relative to control data obtained at the same time points. Each bar represents pooled data from three to six animals performed in triplicate. The vertical bars represent SEM. *p < 0.05 (ANOVA, one-way analysis of variance) represents significant differences from control values obtained in the absence of test drugs; ^#^*p* < 0.05 (ANOVA, one-way analysis of variance) represents significant differences compared to the CTRL group

Figure 5 shows that the enzymatically stable adenosine analogue, NECA (1–30 μM), concentration-dependently increased cell viability and/or growth (Fig. 5A) and type I collagen (Fig. 5B) production by CFs obtained from the RV of both CTRL and MCT-treated rats. Yet, NECA-induced effects were much more evident (*p* < 0.05) in cultured cells isolated from rats with PAH. The pro-fibrotic effect of NECA (1–30 μM) was more evident with the time of the cells in culture (days 21 and 28 > days 7 and 14). Under the present experimental conditions, NECA had an outstanding contribution to CFs viability/proliferation (MTT assay) (Fig. 5A) compared to type I collagen production (Sirius Red assay) by the same cells (Fig. 5B). Given that both 10 and 30 μM NECA yield similar effects, the 10-μM concentration was selected for subsequent experiments.

**Fig. 6:**
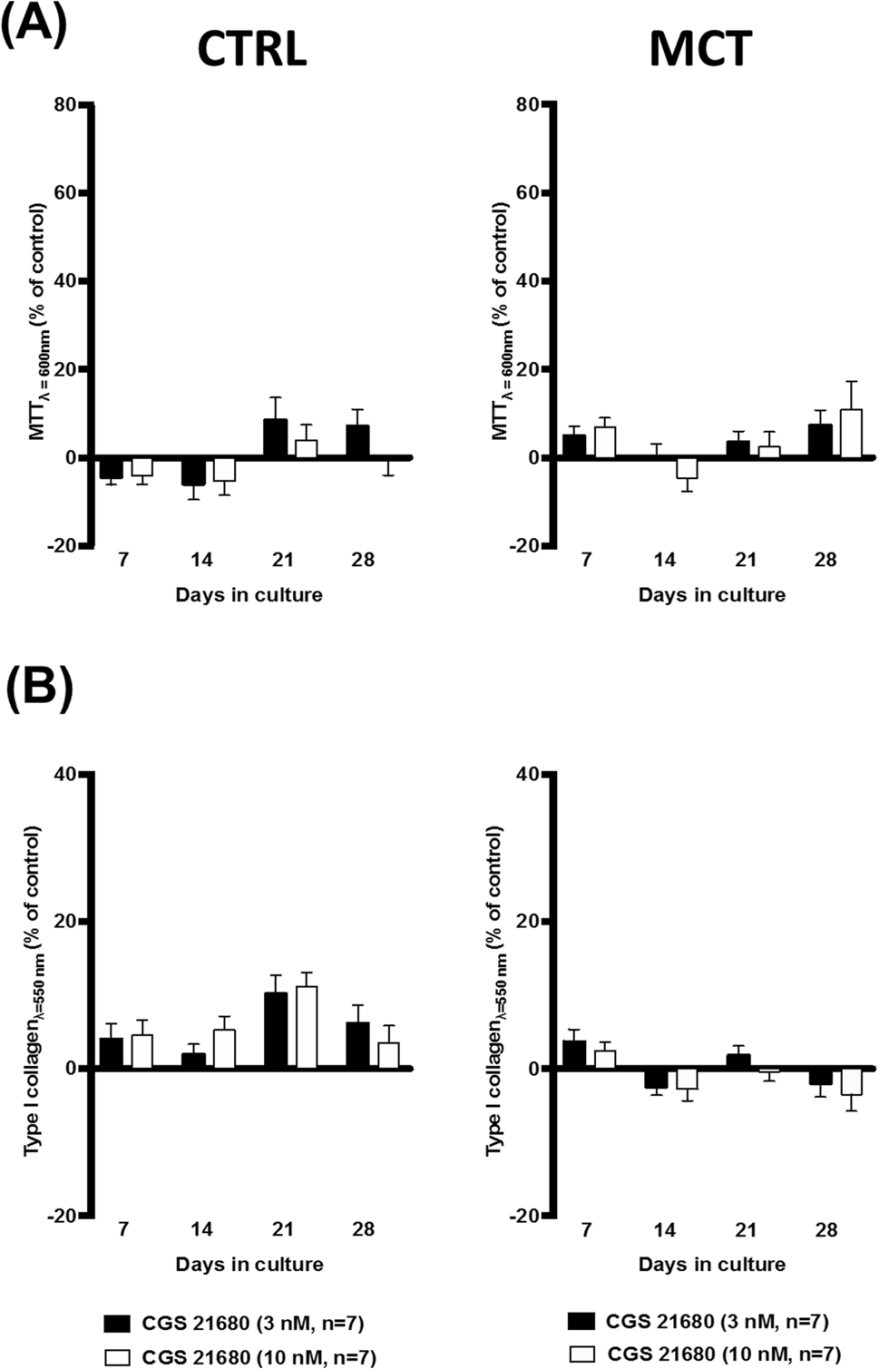
Effects of the selective A_2A_ receptor agonist, CGS216980 (3–10 nM), on cell viability/growth and type I collagen production by cardiac fibroblasts (CFs) from the RV of CTRL and MCT-treated rats. The ordinates represent CGS216980 (3–10 nM)-induced changes in cell growth (MTT assay, **A**) and type I collagen production (Sirius Red assay, **B**) compared to the control situation using the same cell batch in the absence of the A_2A_AR agonist at culture days 7, 14, 21, and 28. Zero represents the similarity between the two values (CGS216980 vs. control); positive and negative values represent facilitation or inhibition of either cell growth or type I collagen production relative to control data obtained at the same time points. Each bar represents pooled data from 7 animals; 3 to 4 replicas were performed for each experiment. The vertical bars represent SEM. *p < 0.05 (ANOVA, one-way analysis of variance) represents significant differences from control values obtained in the absence of test drugs

In contrast to NECA, the potent and selective A_2A_AR agonist CGS21680 (EC_50_ = 1.48–180 nM) had minimal effects on CFs viability/proliferation (MTT assay) (Fig. 6A), as well as on type I collagen production (Sirius Red assay) (Fig. 6B). This was observed even when CGS21680 was used in the 3 to 10 nM concentration range that is known to be effective in other mesenchymal-originated cells, like subcutaneous fibroblasts and bone-marrow osteoblast progenitors [12, 22].

**Fig. 7:**
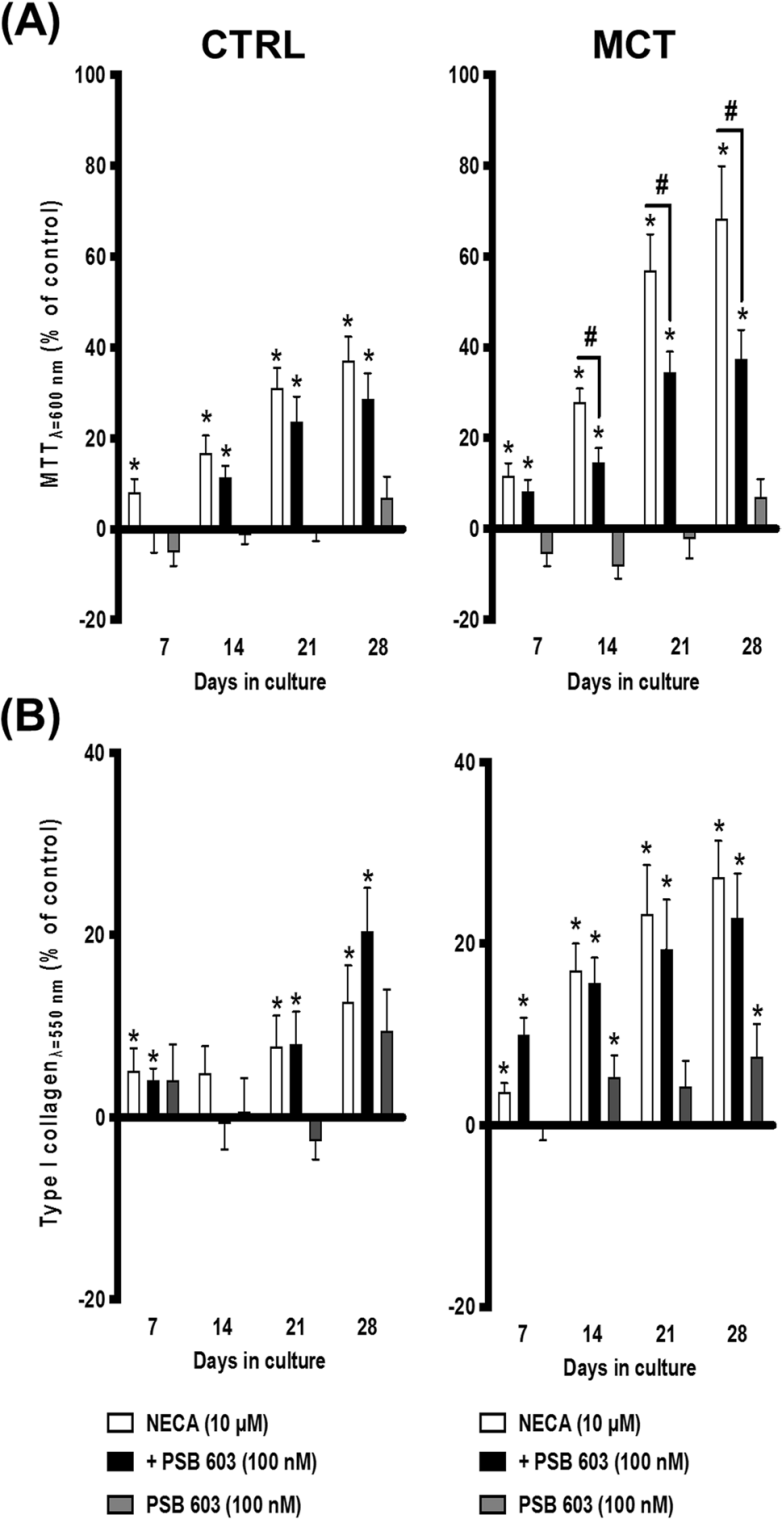
Selective A_2B_ receptor blockage with PSB603 (100 nM) attenuates NECA-induced overgrowth of cardiac fibroblasts (CFs) from the RV of MCT-treated rats. NECA (10 μM) with or without PSB603 (100 nM) was incorporated in culture media throughout the whole assay. The ordinates represent NECA- and/or PSB603-induced changes in cell growth (MTT assay, **A**) and type I collagen production (Sirius Red assay, **B**) compared to the control situation using the same cell batch in the absence of test drugs at culture days 7, 14, 21, and 28. Zero represents the similarity between the two values (drug vs. control); positive and negative values represent facilitation or inhibition of either cell growth or type I collagen production relative to control data obtained at the same time points. Each bar represents pooled data from 5 (CTRL) and 6 (MCT) animals; 3 to 4 replicas were performed for each experiment. The vertical bars represent SEM. *p < 0.05 (ANOVA, one-way analysis of variance) represents significant differences from control values obtained in the absence of test drugs; ^#^*p* < 0.05 (ANOVA, one-way analysis of variance) represents significant differences compared to the effect of NECA alone

**Fig. 8:**
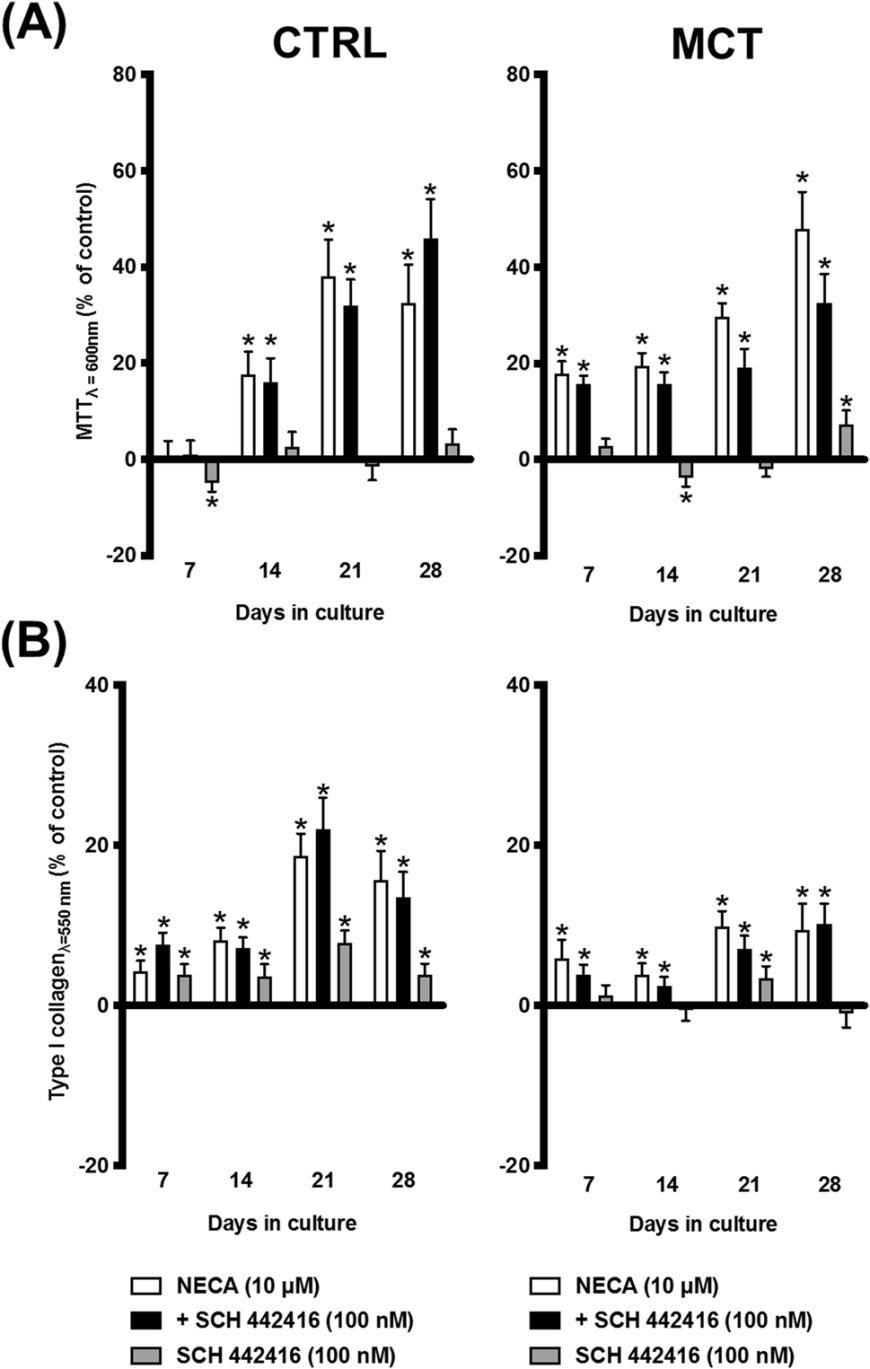
Selective A_2A_ receptor blockage with SCH442416 (100 nM) did not modify NECA-induced overgrowth of cardiac fibroblasts (CFs) from the RV of MCT-treated rats. NECA (10 μM) with or without SCH442416 (100 nM) was incorporated in culture media throughout the whole assay. The ordinates represent NECA- and/or SCH442416-induced changes in cell growth (MTT assay, **A**) and type I collagen production (Sirius Red assay, **B**) compared to the control situation using the same cell batch in the absence of test drugs at culture days 7, 14, 21, and 28. Zero represents the similarity between the two values (drug vs. control); positive and negative values represent facilitation or inhibition of either cell growth or type I collagen production relative to control data obtained at the same time points. Each bar represents pooled data from 8 animals; 3 to 4 replicas were performed for each experiment. The vertical bars represent SEM. *p < 0.05 (ANOVA, one-way analysis of variance) represents significant differences from control values obtained in the absence of test drugs

NECA exhibits the highest affinity for the A_2B_AR subtype among all other adenosine analogues [35]. Considering that, despite this, it may also bind to other P1 adenosine receptors present in CFs, we set to investigate the involvement of the A_2B_AR using the selective A_2B_AR antagonist PSB603 (100 nM), which displays >17,000-fold selectivity for A_2B_AR over other adenosine receptors [26]. Co-application of PSB603 (100 nM) together with NECA (10 μM) attenuated (*p* < 0.05) the effect of this adenosine receptor agonist on cell viability and/or proliferation of CFs isolated from the RV of MCT-treated rats (Fig. 7A), but no effects were observed in type I collagen production by the cells of these animals (Fig. 7B) nor of CTRL rats. The selective A_2A_AR blocker, SCH442416 (10 nM) [27], did not modify the effects of NECA (10 μM) on CFs viability/proliferation (Fig. 8A) and type I collagen production (Fig. 8B), under the same experimental conditions. Given these findings, one cannot exclude the synergistic participation of less dominant P1 receptor subtypes, namely A_1_AR and A_3_AR, in the A_2B_AR-mediated promotion of type I collagen production by CFs of MCT-treated rats.

## Discussion

We report here, for the first time, that activation of the adenosine A_2B_AR subtype increases the metabolic activity and/or the proliferation and type I collagen production by cardiac myofibroblasts isolated from the RV of healthy rats. More importantly, we provide evidence showing that these effects are bolstered in CFs overexpressing the A_2B_AR obtained from animals presenting mild to moderate signs of PAH. Selective blockage of the A_2B_AR activation with PSB603 attenuated NECA-induced increases in cell viability/proliferation of CFs isolated from MCT-induced PAH animals, without affecting the type I collagen production by the same cells.

PAH is a putatively fatal disorder characterized by increased pulmonary vascular resistance, which if left untreated rapidly progresses to right ventricular failure, irreversible dysrhythmias, and death [1]. Besides pulmonary vascular abnormalities, cardiac maladaptive remodelling (e.g., fibrosis), vasoconstriction, inflammation, and thrombosis are also common features of this progressive disorder. Currently approved drug treatments for PAH focus primarily on reducing pulmonary vascular resistance through the use of prostacyclin analogues, endothelin-1 receptor antagonists, phosphodiesterase-5 inhibitors, and soluble guanylate cyclase activators. Yet, these interventions have a limited impact on myocardial remodelling [3, 4]. This gap in our knowledge paves the way for novel therapeutic approaches to specifically target the pathological remodelling process evolving in the myocardium of PAH patients, as these changes critically contribute to the fatal outcomes associated with this disease condition.

The natural course of RV remodelling secondary to PAH is characterized by an early compensatory phase comprising RV hypertrophy and improvement of systolic parameters, which help to preserve the cardiac output, with minimal fibrosis [1]. If the pressure overload is maintained, the RV enters into a decompensated phase resulting in a gradual decline of the RV systolic pressure and, consequently, of the cardiac output, which is accompanied by extensive myocardial fibrosis and dilation of cardiac chambers [36]. Several mechanisms may contribute to the shift from a compensated to a decompensated state, but myocardial fibrosis certainly represents one of the most relevant [37].

The MCT-induced PAH model was selected due to its simplicity and reproducibility, including the ability to induce neuroendocrine and inflammatory activation that is required for rapid progression to RV failure [38]. In the present study, PAH animals grew slowly (gained less body weight) compared to their CTRL littermates from the post-injection day 7 onwards (Fig. 1A), which is aligned with previous findings in the literature [39, 40]. The morphometric analysis revealed an increase in Fulton’s index (Fig. 1B) suggesting that MCT-treated rats exhibited RV hypertrophy [39, 40]. The histological assessment revealed that the cross-sectional area of RV cardiomyocytes was enlarged in MCT-treated rats compared to the CTRL group (Fig. 1C and 1E) [41, 42]. Overall, data demonstrate that a single injection of MCT caused mild to moderate pulmonary pressure overload leading to RV hypertrophy, which is a hallmark of PAH [7, 43].

Despite the aforementioned PAH features, the presence of RV myocardial fibrosis in the MCT-induced animal model has been a matter of debate in the literature. Failure to demonstrate interstitial or replacement fibrosis in MCT-treated rats assessed by the deposition of type I collagen has been claimed by some research groups [44–47]. In our hands, we observed a threefold increase in vimentin-positive CFs infiltrating the RV myocardium of MCT rats compared with healthy controls (Fig. 3B). The overgrowth of CFs in the RV myocardium of MCT-treated animals occurred in parallel with the hypertrophy of cardiomyocytes revealed as increases in the cross-section area of the cells vis-a-vis those obtained from healthy controls (Fig. 1C and 1E). Surprisingly, the Picro-Sirius Red assay detected minimal amounts of collagen in the myocardium 21 days after the MCT injection (Fig. 1D and 1F). This trend may be owed to the belated synthesis of type I collagen compared to the production of other ECM proteins (e.g., type III collagen) [48], in addition to early-to-intermediate stage features of myocardial remodelling comprising CFs overgrowth rather than collagen deposition (see above). It, thus, appears that in this animal model of PAH, the RV remodelling primarily involves cardiomyocyte hypertrophy, CFs overgrowth, and the myofibroblastic transformation of these cells towards the expression of α-SMA and DDR2 [49], rather than collagen deposition (fibrosis) in the myocardial interstitial spaces [7, 47, 50]. The incapacity to target CFs proliferation and these cells’ differentiation into activated myofibroblasts may explain why currently available medications fail to effectively address the RV remodelling and, thus, the inexorable progression to RV failure associated with PAH [51].

CFs are the most abundant cell type in the heart [52], which along with cardiomyocytes play prominent roles in defining cardiac structure and function [53]. Given their abundance, strategic location within the cardiac interstitium, and ability to modulate their own and other cells’ functions (e.g., cardiomyocytes, inflammatory cells, and endothelial cells), CFs play a sentinel role to detect myocardial injury states, as well as to transmit information to initiate reparative responses and to coordinate myocardial remodelling [54]. CFs proliferation and differentiation into myofibroblasts are critical features underlying cardiac fibrosis, which is one of the main pathological findings of heart failure. Cardiac fibrosis not only diminishes cardiac reserve but also increases the susceptibility to life-threatening dysrhythmias. Hence, from a clinical perspective, elucidating the mechanism(s) behind CFs proliferation and/or differentiation holds paramount importance to uncover novel pharmacological targets for PAH treatment.

Among the various autocrine/paracrine signalling messengers originating from pathologically stressed cells, adenine nucleotides and nucleosides emerge as important players in the regulation of CFs’ growth and differentiation [12–14]. Adenosine is a retaliatory metabolite playing important roles in myocardial reperfusion, hypertrophy, and remodelling. Extracellular adenosine levels rise dramatically in response to metabolic stress; the nucleoside can be released as such, via equilibrative nucleoside transporters, or can originate as a consequence of the extracellular ATP breakdown, via the ectonucleotidase cascade [55]. Adenosine exerts its biological effects by binding to P1 receptors (A_1_AR, A_2A_AR, A_2B_AR, and A_3_AR) [8]. Under pathological conditions, high extracellular levels of the nucleoside favor activation of low-affinity A_2B_AR, which are normally silent under physiological conditions [21, 22, 56]. Emerging evidence indicates that pathological conditions favor A_2B_AR-mediated “biased” signalling cascades depending on various factors, namely receptors cell trafficking and localization, post-translational modifications, and promiscuous coupling to other effector proteins (reviewed in [9]). Previous studies suggest a detrimental impact of the A_2B_AR subtype in chronic lung diseases, particularly concerning lung and cardiac fibrosis [57, 58]. Despite all adenosine receptor subtypes are represented in rat CFs, the A_2B_AR is the most abundant, followed by A_2A_AR and A_1_AR subtypes, with minimal amounts detected for the A_3_AR [15, 59]. Regardless of their presence, the participation of adenosine receptors in the control of CFs growth, differentiation, and cardiac fibrosis is far from being completely understood (reviewed in [60]) and, so far, few studies investigated their role in the context of PAH [61–64].

CFs isolated from MCT-treated rats are metabolically more active and/or grow faster and overexpress A_2B_AR compared to cells obtained from healthy controls. Upregulation of the A_2B_AR was also observed in CFs from human patients with LV dysfunction secondary to valvulopathies [65]. Others have shown that CFs isolated from RV of MCT-induced PAH rats proliferate more than the cells from CTRL littermates [61]; these authors implicated in this mechanism calcium-dependent (SOCE/CaMKII) and calcium-independent (ERK1/2) pathways. Epigenetic reprogramming of mitochondrial metabolic pathways also seems to participate in CFs overgrowth observed in the RV of MCT-treated animals [62, 63]. In addition, the involvement of cyclic GMP-dependent pathways on collagen production by CFs isolated from the RV of mice subjected to pulmonary artery banding has been demonstrated, which further validates the use of medications, like sildenafil and riociguat, in the treatment of PAH [64].

The immunoblot analysis of cultured CFs extracts performed in our study detected two protein isotypes (see Fig. 4): one around 37 kDa (the predicted molecular weight of the A_2B_AR) and another with a higher molecular mass (~45 kDa). The naturally occurring 37 kDa protein isotype is the main contributor to the A_2B_AR enrichment in CFs isolated from the RV myocardium of MCT-treated rats. At this point, one may only speculate that the higher molecular mass (~45 kDa) band existing both in CTRL and MCT-treated CFs might correspond to an A_2B_AR protein species affected by post-translational modifications, most commonly due to glycosylation [66]. This assumption, as well as the pathophysiological meaning of these findings, certainly deserves to be fully elucidated in the future.

Our data show that the enzymatically stable adenosine analogue, NECA, increases the metabolic activity and/or the proliferation and type I collagen production by CFs obtained from both CTRL and MCT-treated rats, but these effects were much more evident in cells isolated from pressure-overloaded RV of rats with PAH. Here, NECA was used within a concentration range (1–30 μM) known to activate the A_2B_AR subtype (EC_50_ of 2 μM) [67–69]. The involvement of the A_2B_AR subtype was confirmed by blocking NECA-induced cell viability/proliferation with the selective A_2B_AR antagonist, PSB603, whereas the A_2A_AR antagonist, SCH442416, had no effect under the same experimental conditions. The potent and selective A_2A_AR agonist, CGS21680 (EC_50_ = 1.48–180 nM), failed to modify growth and type I collagen production by CFs isolated from both control and MCT-treated rats, even when applied in concentrations (3 nM and 10 nM) high enough to increase the proliferation of other mesenchymal-originated cells [12, 22]. These findings suggest a dominant participation of the A_2B_AR subtype in the pro-fibrotic effect of NECA, while minimizing the involvement of the A_2A_AR subtype in this mechanism, regardless of data approaching pulmonary microvessel remodelling and hypertrophy suggesting that targeting the latter receptor may be beneficial for treating PAH and concomitant RV failure (reviewed in [16]). The lack of A_2A_AR-mediated effects in moderate to severe disease stages is not surprising, since rats with PAH-induced RV failure exhibit reduced A_2A_AR levels in the myocardial tissue [16]. Moreover, the pro-fibrotic effect of the A_2B_AR subtype in the RV of PAH rats replicates that were verified in the lung [70] and kidney [71] preparations, thus strengthening its role in fibrotic processes.

Interestingly, selective blockage of A_2B_AR activation with PSB603 attenuated NECA-induced increases in cell viability/proliferation of CFs from the RV of PAH rats with a minor (if at all) effect on type I collagen production by these cells. Parallel histopathological assays carried out in the same myocardial samples fully agree with data obtained in isolated CFs, given that the RV of MCT-treated rats displays extensive interstitial infiltration by triple-positive vimentin/DDR2/α-SMA CFs adjacent to hypertrophied cardiomyocytes, but shown only small amounts of collagen in the same regions as demonstrated by the Picro-Sirius Red staining. Thus, one may speculate that endogenously generated adenosine, acting via A_2B_AR, may contribute to CFs growth and myofibroblastic differentiation of these cells with only a minimal influence on ECM deposition at this disease stage. Despite the low-affinity A_2B_AR subtype has been extensively implicated in multiple pathological conditions [72], supporting its pivotal role in excessive CFs growth in the RV myocardium of PAH rats, one cannot discount the participation of other NECA-sensitive P1 receptors in collagen production. Considering that NECA-induced collagen production by cultured CFs of PAH rats transcended that obtained in CTRL animals, yet no prevention of the NECA effect was obtained upon blocking A_2B_AR (with PSB603) and A_2A_AR (with SCH442416), one may hypothesize the participation of minority A_1_AR and/or A_3_AR in this endeavor [15, 59]. Despite no specific involvement of A_1_AR and/or A_3_AR has been addressed in the context of PAH has not been addressed, evidence exists that the expression and signalling via A_1_AR and/or A_3_AR may increase fibrosis in several pathological conditions [65, 73–76].

The A_2B_AR subtype has been previously implicated in experimental cardiac fibrosis induced in mice [77], rats [78], and humans [78, 79]. In CFs from healthy human subjects, NECA (10 μM) increased the expression of α-SMA and α-1 procollagen, as well as the production of soluble collagen; these effects were effectively blocked by GS-6201 (100 nM), a selective A_2B_AR antagonist [78, 79], which was also able to attenuate pulmonary fibrosis [70]. Similar to the antiproliferative activity of the A_2B_AR antagonist, PSB603, on CFs from the RV of PAH rats, Zhang and co-workers showed that GS-6201 could reduce myocardial fibrosis in a rat model of myocardial infarction [78]. In another study, Karmouty-Quintana et al. showed that the A_2B_AR activation promotes endothelin-1 and IL-6 release from endothelial cells and pulmonary artery myocytes, thus contributing to vessel wall remodelling and PAH progression [80]. Despite these findings, the A_2B_AR-mediated pro-fibrotic activity in the heart is not consensual [67, 81–83]. Some authors claim that adenosine inhibits collagen synthesis [84] and mitogenesis [67] of CFs in adult rats, thus supporting the anti-fibrotic action of the nucleoside via A_2B_AR activation in an “*in vivo*” animal model of myocardial infarction [83]. The A_2B_AR was also involved in the inhibition of ET-1-induced cardiac fibrosis [85]. Likewise, overexpression of A_2B_AR decreased collagen synthesis, while silencing the A_2B_AR gene with a siRNA had the opposite effect [82]. Nevertheless, a controversy was installed in the same work as authors found that increasing concentrations of the enzymatic-stable adenosine analogue, NECA, favored, instead of preventing, collagen synthesis from A_2B_AR overexpressing CFs [82], which fully agrees with our findings using primary CFs cultures from adult rats with PAH. The cardiac anti-fibrotic activity of the hybrid molecule, VCP746, is even more complicated to interpret, as this compound displays A_1_AR agonist properties while also binding bivalently with high affinity to A_2B_AR [86].

Most studies reporting protective roles of the A_2B_AR in PAH have focused on its involvement in vascular remodelling, rather than on its role in subsequent RV myocardium overload and fibrosis; these studies often conclude on the A_2B_AR enrolment by blocking this receptor activation with selective antagonists or gene silencing approaches (for a review, see, [9]). Under such conditions, elimination of the A_2B_AR tonus is dependent on extracellular adenosine production/accumulation, which is a difficult issue to achieve given that the A_2B_AR displays a low affinity for the nucleoside and, therefore, this receptor activation requires high extracellular adenosine concentrations. These controversies may reflect our incomplete understanding of the mechanism(s) driving cardiac remodelling in response to RV pressure overload in PAH. Variations in the experimental conditions (e.g., stage of the disease condition before culturing the cells; neonatal vs. adult cell cultures; the number of cell passages; stiffness of culture surfaces; and serum-starvation conditions) may additionally affect the phenotype, growth, differentiation, and function of cultured CFs. For instance, increasing the number of cell passages decreases CFs proliferation and leads to morphological changes associated with cell senescence [87]. Non-standardized drug application protocols may also introduce confounding effects; for instance, NECA inhibited proliferation and collagen synthesis when CFs were treated every 24 h for a maximum of 4 days [67]. In our study, we used rats with mild to moderate PAH before cell isolation and only the first-cell subcultures were assayed to avoid phenotypic de-differentiation artifacts. CF cultures were allowed to grow for 28 days in the presence of test drugs, which was directly compared with the control situation using the same cellular batch but without test drugs. Due to limited information regarding the role of A_2B_AR on CFs proliferation and differentiation during the timeframe required for “*in vivo*” cardiac remodelling, we consider our results obtained in 28-day cultures derived from the RV myocardium of PAH animals a proxy of the real cells exposure to the nucleoside. Nevertheless, the current study has also potential limitations, since (i) we only assessed MCT-induced animals presenting mild to moderate PAH signs, and did not investigate (ii) how signalling events downstream A_2B_AR activation could modulate cardiac fibrosis in PAH, nor (iii) did we explore the putative involvement of minority A_1_AR and/or A_3_AR in the pro-fibrotic effect of NECA.

In conclusion, this study provides the first evidence that adenosine, via A_2B_AR activation, plays a pivotal role to increase cell viability/proliferation and myofibroblast differentiation of CFs from pressure-overloaded RV myocardium of rats with mild to moderate PAH. These findings highlight the putative therapeutic relevance of targeting CFs viability/growth and differentiation by blocking the A_2B_AR activation to prevent cardiac remodelling and mitigate right heart failure in PAH patients. The clinical implications of our study extend beyond the RV myocardium, as targeting A_2B_AR activation might also have a beneficial impact on pulmonary vascular remodelling and lung fibrosis underlying this yet incurable disorder (see [9, 16]).

## Abbreviations

A: Absorbance
A_2A_AR: Adenosine A_2A_ receptor
A_2B_AR: Adenosine A_2B_ receptor
BSA: Bovine serum albumin
CGS21680: 4-[2-[[6-Amino-9-(nethyl-β-D-ribofuranuronamidosyl)-9H-purin-2-yl]amino]ethyl] benzene-propanoic acid hydrochloride
CTRL: Control
DAPI: 4′-6-Diamidino-2-phenylindole
DMSO: Dimethyl sulfoxide
ECM: Extracellular matrix
FBS: Fetal bovine sérum
MCT: Monocrotaline
MTT: 3-[4,5-Dimethylthiazol-2-yl]-2,5-diphenyltetrazolium bromide
NECA: 5′-*N*-ethylcarboxamidoadenosine
PAH: Pulmonary arterial hypertension
PBS: Phosphate-buffered saline
PFA: Paraformaldehyde
PSB603: 8-[4-[4-(4-Chlorophenzyl)piperazide-1-sulfonyl)phenyl]]-1-propylxanthine
RV: Right ventricle
SCH442416: 2-(2-Furanyl)-7-[3-(4-methoxyphenyl) propyl]-7H-pyrazolo[4,3-e][1,2,4]triazolo[1,5-*c*]pyrimidin-5-amine
